# The Centre for Healthy Weights—Shapedown BC: A Family-Centered, Multidisciplinary Program that Reduces Weight Gain in Obese Children over the Short-Term

**DOI:** 10.3390/ijerph8124662

**Published:** 2011-12-15

**Authors:** Constadina Panagiotopoulos, Rebecca Ronsley, Mohammed Al-Dubayee, Rollin Brant, Boris Kuzeljevic, Erin Rurak, Arlene Cristall, Glynis Marks, Penny Sneddon, Mary Hinchliffe, Jean-Pierre Chanoine, Louise C. Mâsse

**Affiliations:** 1 Department of Pediatrics, University of British Columbia, Vancouver, British Columbia, V6T 1Z4, Canada; Email: erinrurak@gmail.com (E.R.); jchanoine@cw.bc.ca (J.-P.C.); lmasse@cfri.ubc.ca (L.C.M.); 2 British Columbia Children’s Hospital, Vancouver, British Columbia V6H 3V4, Canada; Email: Arlene.Cristall@phsa.ca (A.C.); PSneddon@cw.bc.ca (P.S.); mhinchliffe@cw.bc.ca (M.H.); 3 Child & Family Research Institute, Vancouver, British Columbia, V5Z 4H4, Canada; Email: rollin@stat.ubc.ca (R.B.); bkuzel@cw.bc.ca (B.K.); 4 University of Toronto, Toronto, Ontario M5S 1A1, Canada; Email: rebecca.ronsley@gmail.com; 5 Department of Pediatrics, King Saud Bin Abdulaziz University for Health Sciences, Riyadh, Saudi Arabia; Email: dubayeem@yahoo.com; 6 Department of Statistics, University of British Columbia, Vancouver, British Columbia, V6T 1Z4, Canada; 7 Nanaimo Regional General Hospital, Nanaimo, British Columbia, V9S 2B7, Canada; Email: glynis.marks@viha.ca; 8 Department of Family Practice, University of British Columbia, Vancouver, British Columbia, V6T 1Z4, Canada; 9 School of Population and Public Health, University of British Columbia, Vancouver, British Columbia, V6T 1Z4, Canada

**Keywords:** obesity, weight loss, BMI reduction, children, prevention, treatment, intervention

## Abstract

The objective was to conduct a program evaluation of the Centre for Healthy Weights—Shapedown BC (CHW-SB), a family-centered, multidisciplinary program for obese children, by assessing the change in weight trajectories from program intake to completion. Secondary outcomes included changes in clinical, biochemical and psychological parameters, and in physical activity (PA) levels. The CHW-SB program was evaluated over 10 weeks. Data collection included anthropometric, metabolic, PA and psychological measures. Longitudinal mixed effects regression was performed to evaluate weight change from Phase 1 (before program on waitlist) to Phase 2 (during program). 238 children <18 years of age were referred to the program of which 119 were eligible for participation. There was a significant decrease in weight trajectory in children following program entry. Participants experienced an average .89% monthly increase before program entry, compared to a .37% monthly decline afterwards, a drop of 1.26% (*p* < 0.0001, 95%CI 1.08 to 1.44). *z*BMI (2.26 ± 0.33 to 2.20 ± 0.36, *p* < 0.001), waist circumference (99 ± 15.7 to 97 ± 16 cm, *p* < 0.0001) and fasting insulin (137 ± 94.8 to 121 ± 83.4 pmol/L, *p <* 0.001) also decreased in participants who attended the final visit. Significant improvements were seen in all measures of PA, self-concept, and anxiety. CHW-SB, a government-funded program, is the first obesity-treatment program to be evaluated in Canada. While short-term evaluation revealed significant improvements in adiposity, PA, and psychological measures, the lack of full follow-up is a limitation in interpreting the clinical effectiveness of this program, as drop-out may be associated with lack of success in meeting program goals. These data also emphasize the need for ongoing evaluation to assess the long-term implications of this unique program and ultimately optimize utilization of governmental resources.

## 1. Introduction

Childhood obesity is a serious public health concern worldwide [1,2,3]. In Canada, the prevalence of overweight and obesity in children has increased over recent decades. In 2004, it was estimated that over 25% of Canadians aged 2 to 17 years were overweight or obese, which is a significant increase from 15% recorded in this age group in 1979 [4]. Given this trend towards weight gain in children [5], and the mounting evidence that overweight and obesity may track through to adulthood [6,7,8,9,10], it is clear that childhood obesity is a public health crisis that must be addressed. 

Further to the growing body of literature showing increases in obesity prevalence, there are also compelling data demonstrating a positive relationship between obesity and risk for future chronic disease. Specifically, an elevated body mass index (BMI) in children greatly increases the likelihood of developing type 2 diabetes, hypertension, hyperlipidemia, nonalcoholic fatty liver disease, orthopedic complications and sleep apnea [11,12,13,14,15,16]. Furthermore, an elevated BMI is associated with the metabolic syndrome, which includes a constellation of cardiometabolic risk factors such as central adiposity, dyslipidemia, dysglycemia and elevated blood pressure [11,17]. 

It is estimated that as many as 30% of obese adolescents have the metabolic syndrome [13]; a concerning observation as these children have an increased risk of developing diabetes mellitus and cardiovascular disease in adulthood [14,15,16,18]. In addition to developing serious medical co-morbidities, overweight and obese children are also at a higher risk of experiencing psychological problems including mood and anxiety disorders [19,20]. Furthermore, overweight children may suffer from early discrimination [21,22] and an overall poorer quality of life [23]. The current burden of childhood obesity on both medical and psychological well-being makes it important to develop effective interventions in this population. 

Currently, options for long-term management of obesity include programs targeting behavior surrounding diet and exercise, pharmacological agents, and bariatric surgical approaches. In the pediatric population, lifestyle modification interventions should be the first line of treatment when dealing with this problem, given the invasiveness of other approaches [24]. Such interventions must specifically focus on teaching the family effective strategies to improve their dietary intake, to increase their levels of physical activity, and to decrease their sedentary behavior. 

Lifestyle and behavior modification approaches have been found to be efficacious to treat childhood obesity, at least in the short-term [25,26,27]. Results from a recent meta-analysis suggest that, in randomized control trials, interventions in the pediatric population have resulted in an 8.2% to 8.9% decrease in weight compared to controls who have observed an increase in weight of 2.1% to 2.7% [25]. Therefore, lifestyle behavioral interventions compared to standard care can produce clinically significant reductions in weight for children and adolescents [28]; however, these programs have yet to be evaluated in Canada once implemented in a clinical setting.

The objective of this study was to evaluate the Centre for Healthy Weights—Shapedown BC (CHW-SB) familial intervention program targeted at overweight and obese children by assessing the change in weight trajectories from program intake date to program completion date. We hypothesized that the CHW-SB program would decrease the slope of the weight gain trajectory in children over the course of the 10-week program. Our secondary outcomes included changes in clinical, biochemical and psychological parameters, as well as changes in physical activity levels in program participants.

## 2. Methods

### 2.1. Study Design

The protocol was approved by both the University of British Columbia Clinical Research Ethics Boards and the Children’s & Women’s Research Review Committee. Parents provided informed written consent and children provided assent. This study was a registered clinical trial (NCT00564798) designed as a prospective program evaluation of a cohort of participants where children were assessed from date of intake to the CHW-SB program (described in detail below) until date of completion of the 10-week program. Participants waited a median of 87 days (range: 12 to 272) to participate in the CHW-SB program. They were measured at the intake visit and again at the time of starting the program, which made it possible to assess weight trajectories changes before the program intervention compared to during the program. By taking advantage of this practical situation, we were able to obtain a waitlist control group for comparison purposes without delaying access to treatment.

### 2.2. The Program

The CHW-SB program was based on the *Shapedown* program developed in the United States as a family-centered, multidisciplinary program that includes cognitive, behavioral, affective and interactional techniques for obese children and their families. In a pilot evaluation conducted by Mellin *et al.*, *Shapedown* participants demonstrated improvements in weight, self-esteem, weight-management knowledge and depression [29]. After the success of the *Shapedown* pilot in California [29,30], a modified version of the program was developed to meet the needs of overweight and obese youth seen at British Columbia Children’s Hospital. The CHW-SB program is a family-centered, behavioral weight management program that provides medical and psychosocial assessment, education and support to obese and overweight children and their families. The CHW-SB program was funded by the British Columbia Ministry of Health Services in 2006 through ActNow BC and is the only treatment program for childhood and adolescent obesity in British Columbia [31]. The CHW-SB program is staffed by a multidisciplinary team that includes a registered dietitian, a psychologist, and a physician. This core team provides the comprehensive assessment component for each family referred to the program while the intervention is co-facilitated by the dietitan and the psychologist. The YMCA provides an exercise specialist for each session on a contractual basis. The intervention consists of 10 consecutive weekly group sessions, each two hours in duration. Each group consists of six to 10 families, assembled according to the children’s ages. Each session includes separate child and parent sessions led by the psychologist or dietitian. Each session also includes 30 min of physical activity for the children led by the YMCA fitness instructor that focuses on strength, flexibility and/or endurance with a concurrent discussion for parents led by a psychologist. Examples of exercise session themes include Latin Groove (Zumba Class), Tai-Chi, Hip-Hop, Yoga & Pilates, Tae Kwon Doe, Circuit Training, Sports Drills, Muscle Pump, Kick-Box Fit. The goal of these sessions is to get the kids comfortable with moving their bodies and to link them to an activity within their own community. Sessions conclude with joint family activities including a nutrition theme following Canada’s Food Guide [32] or a psychological theme in conjunction with goal-setting for the next session. Examples of nutrition topics addressed include label reading, healthy snacking, decreasing sugar-sweetened beverage consumption, promoting fibre and fruit and vegetable consumption, portion sizes, and healthy family eating styles. Psychology sessions focus on goal-setting, communication, appropriate limit-setting and expectations, problem-solving and managing challenges, building self-esteem, developing realistic thinking, and dealing with teasing/bullying. After completion of the program, monthly follow-up sessions are offered on an ongoing basis. 

### 2.3. Participants

Subjects were children who were referred to the program by family physicians, community pediatricians, and British Columbia Children’s Hospital pediatric subspecialists between March 2007 and March 2009. Children were eligible for referral if they were 6–17 years old and were either obese (BMI ≥ 95^th^ percentile for age and sex) or overweight (≥ 85^th^ percentile and < 95^th^ percentile) [33] with at least one co-morbidity (including impaired fasting glucose, dyslipidemia, polycystic ovarian syndrome, hypertension, obstructive sleep apnea or orthopedic complications). Furthermore, for children to be eligible to participate, at least one parent or caregiver had to be willing to participate and to be able to read and understand English. Individuals were excluded from the program if they were non-ambulatory, undergoing rigorous medical therapy, or were diagnosed with a severe mental illness. Eligibility was initially assessed by a psychologist via telephone interview and confirmed by an intake assessment by the multidisciplinary team

### 2.4. Data Collection

Table 1 summarizes all measures collected at the various time points. At intake, socio-demographic information (including date of intake, date of enrollment, age, sex and ethnicity), anthropometric measurements (including height, weight, waist circumference, and blood pressure), metabolic profile (including fasting plasma glucose, fasting insulin, total cholesterol, high-density lipoprotein cholesterol, low-density lipoprotein cholesterol, and triglycerides), physical activity assessment, and psychological parameters (including the Child Behavior Checklist and The Beck Youth Inventory) (described below) were collected from each child.

**Table 1:** Summary of data collected by time point.

Assessment		Time Point			
		Intake	Baseline (Week 1)	Weeks 2–9	Program completion (Week 10)
**Social Demographic Information**	Date of Birth	✓			
	Sex	✓			
	Ethnicity	✓			
**Anthropometric Measurements**	Waist Circumference	✓	✓		✓
	Weight	✓	✓	✓	✓
	Height	✓	✓		✓
	Blood Pressure	✓			
**Metabolic Profile**	Fasting Glucose	✓			✓
	Fasting Insulin	✓			✓
	Fasting Lipid Panel *	✓			
**Physical Activity Assessment**	Physical Activity Questionnaire		✓		✓
**Psychological Parameters**	Child Behaviour Checklist	✓			
	Beck Youth Inventory	✓			✓

* Includes total cholesterol, low-density lipoprotein cholesterol, high-density lipoprotein cholesterol and triglycerides.

#### 2.4.1. Anthropometric Measurements

Height was measured at intake, baseline and program completion, and weight was measured at intake, weekly during the 10-week program and at program completion. Both height and weight were measured with participants lightly clothed and without shoes to the nearest 0.1 cm using a Seca portable stadiometer (Seca 214 Portable Stadiometer, Seca North America West, Chino, CA, USA) and to the nearest 0.1 kg using an electronic scale (Conair digital electronic scale, Woodbridge, ON, Canada). The average of the two measurements was recorded for analysis. BMI was calculated using weight (kg)/[height (m)]^2^. BMI *z*-score (BMI standardized for age and sex) was calculated using US Centers for Disease Control and Prevention Growth Curves [34]. Waist circumference was measured at intake, baseline and program completion at the umbilicus at the end of expiration using a non-elastic flexible tape measure and was recorded to the nearest millimeter [35]. In this study, adiposity measures include absolute weight, *z*BMI, and waist circumference. Blood pressure (BP) was measured at intake only and recorded as an average of three measures on the right arm and The National High Blood Pressure Education Working Group pediatric definition of elevated BP (systolic or diastolic blood pressure ≥ 90^th^ percentile for age, sex and height) was used [36].

#### 2.4.2. Metabolic Profile

Laboratory samples were obtained after at least 8 hours of fasting. Fasting glucose and fasting insulin were obtained at intake and 10 weeks and cholesterol, high-density lipoprotein cholesterol (HDL-c), low-density lipoprotein cholesterol (LDL-c) and triglycerides were obtained at intake only. Insulin resistance was calculated using the homeostasis model assessment of insulin resistance (HOMA-IR): [Fasting glucose (mmol/L) × (fasting insulin (pmol/L)/7)]/22.5 [37]. The conversion factor of 1/7 for insulin from pmol/L to mU/L is based on BC Children’s Hospital laboratory participation in external quality assessments for the insulin assay [data not shown].

A pediatric definition of the metabolic syndrome modified from the Third National Cholesterol Education Program (NCEP III) [15], comprising at least any three of the following features: WC ≥ 90^th^ percentile for age and sex [38], TG ≥ 1.7 mmol/L, HDL-c ≤ 1.03 mmol/L, elevated systolic or diastolic blood pressure ≥ 90^th^ percentile for age, sex and height and impaired fasting glucose ≥ 5.6 mmol/L (modified to reflect the 2007 American Diabetes Association definition of IFG) [39] was used. 

#### 2.4.3. Physical Activity Assessment

The physical activity questionnaire (PAQ), modified from Crocker *et al.* [40] was used to assess the participant’s level of physical activity and was administered at baseline and at program completion. PAQ is a self-administered questionnaire that has been validated for use in children [41,42] and is used to determine the amount of physical activity performed in the seven days preceding the evaluation. Parents were available to assist younger children in completing the PAQ where required. The PAQ specifically focuses on moderate-to-vigorous activities that are greater or equal to 2.8 MET (where 1 MET is the metabolic equivalent of energy expanded at rest). We estimated standardized energy expenditure (MET*minutes) by multiplying the minutes obtained in the PAQ with the corresponding MET equivalent for the activities as found in the *Ainsworth Compendium of Physical Activity* [43].

#### 2.4.4. Psychological Parameters

The Child Behavior Checklist (CBC) [44] was completed by the parents at intake. It provides a standardized description of skills, emotional and behavioral problems previously validated for use with children aged 6–18 years old [45]. The emotional and behavioural problems are grouped as Internalizing or Externalizing problems. Internalizing problems include anxiety, depression and withdrawn behaviors. Externalizing problems include those that involve conflict with others, such as rule-breaking and aggressive behaviors. The Beck Youth Inventories - Second Edition (BYI-II) for self-concept, anxiety and depression were completed by the children/teens at intake and program completion. Raw scores were converted to T scores [46] and are described as mean ± SD specific to the subject’s normative group.

### 2.5. Statistical Analysis

The analysis was done using STATA**^®^** 10.1 software (StataCorp LP, Texas, USA) package and PASW^®^ Statistics Version 18 (SPSS Inc., Chicago, USA). All continuous variables were expressed as mean ± SD and paired-samples t-tests were used to assess changes in these variables over the course of the program. Where the data was not normally distributed, a Wilcoxon signed-rank test was used in place of a *t*-test.

Longitudinal mixed effects regression analysis [47] was performed to assess whether weight trajectories changed during participation in the CHW-SB program. This method allows inclusion of all available weight measurements and avoids the inherent bias of including only those that completed the program. The natural logarithm (log) of weight measured at intake and weekly during the program was used as the dependent variable rather than BMI *z*-score since height was only measured at intake, baseline, and evaluation. To test change in slope as a result of participating in the CHW-SB program, the weight measurements were divided into two phases. Phase 1 (intake to baseline) included weight change that occurred while the participants were on the program waitlist. Phase 2 (baseline to evaluation) included weight change that occurred during the 10-week CHW-SB program. Independent variables were sex, age at program entry, phase, and days elapsed from program entry (taking negative values for weights recorded in the intake phase). Trajectory slopes for phases 1 and 2 were compared and served to assess the effect of the CHW-SB program, where a significant change in slope was indicative of a program effect. Slopes are reported as monthly percentage growth for ease of interpretation. A *p-*value <0.01 was considered statistically significant to adjust for multiple comparisons.

## 3. Results

### 3.1. Study Participant Characteristics

**Figure 1:**
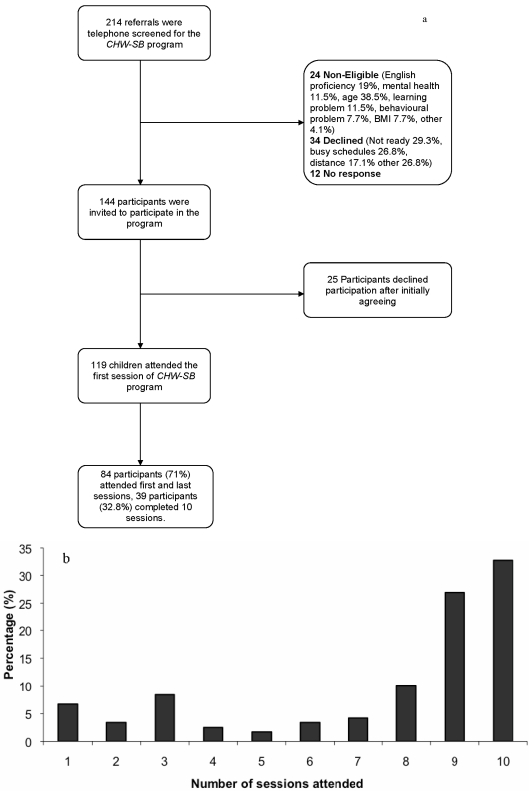
Figure 1 represents the number of sessions that program participants attended. (**a**) Flow Chart of Study Participants, and (**b**) Number of Sessions Attended.

Of the 214 children referred to the CHW-SB program, 42.2% were referred by family physicians, 40.2% by general pediatricians, and 17.6% by pediatric sub-specialists. Individuals declined participation due to geographic location or motivation and readiness barriers. The flow of subjects through the study is presented in Figure 1a. The proportion of children that attended each session is presented in Figure 1b. Of the 138 obese and 6 overweight youth invited to participate in the CHW-SB program, 119 of the participants attended the first session and 84 participants (70.6%) attended both the first and last sessions. A total of 32.8% completed all 10 sessions.

**Figure 2:**
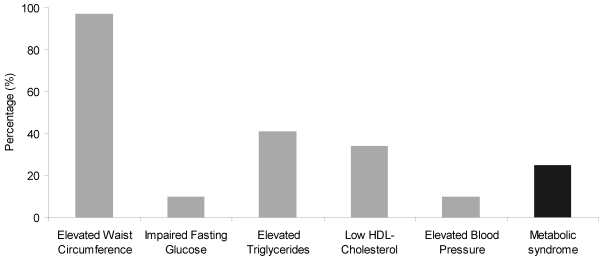
Prevalence of metabolic syndrome and its components at intake.

Baseline characteristics of the program participants are shown in Table 2. At intake, study participants had a mean BMI of 30.9 ± 6.20 kg/m^2^, and the mean BMI *z*-score was 2.3 ± 0.33. The mean waist circumference was 98.5 ± 15.70 cm, and 97.5% of the children had a waist circumference ≥90^th^ percentile. The prevalence of the metabolic syndrome and its individual components are presented in Figure 2. At intake, 27.7% of the study population met diagnostic criteria for the metabolic syndrome. 

**Table 2:** Baseline characteristics at intake.

Characteristics	*CHW-SB* participants *N* = 119		
Age (years)	11.6 ± 2.6		
Male sex (%)	57		
Weight (kg)	76.7 ± 26.8		
Height (cm)	154.5 ± 14.7		
Body Mass Index (kg/m^2^)	30.9 ± 6.20		
BMI *z-*score	2.3 ± 0.33		
Waist circumference (cm)	98.5 ± 15.70		
**Ethnicity: (%)**			
Caucasian	55.6		
Asian	17.6		
Indian/Pakistani	13.4		
Arab	4.2		
African American	1.7		
South American	0.8		
First Nations	0.8		
Other *	5.9		
**Child Behavior Checklist**	**Normal**	**Borderline**	**Clinical**
Internalizing Problems (%)	41.8	9.1	49.1
Externalizing Problems (%)	69.1	11.8	19.1

All data are expressed as mean ± SD unless otherwise specified; * Other ethnicities include: Caribbean and one parent Caucasian + second parent Asian, African American, or Indian/Pakistani.

In addition, psychological data collected at intake revealed that 49.1% and 19.1% of participants, respectively, presented with Internalizing and Externalizing problems that fell within the Clinical range (>97^th^ percentile).

### 3.2. Change in Weight Trajectory

**Figure 3:**
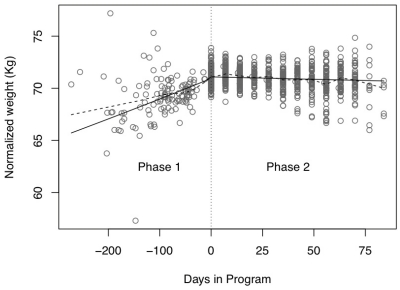
Weight Trajectory from Intake to Baseline (Phase 1) and to Program Completion (Phase 2). Baseline Visit= Day zero; 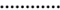 = Non-parametric smooth of the data points; 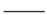 = Overall prediction line from the mixed model effect; Normalized weight = log(weight); Phase 1: Intake to program baseline; Phase 2: Baseline to program evaluation.

Figure 3 summarizes the change in weight before participation in CHW-SB program and the change in weight after participating in the program. Participants experienced an average .89% (95%CI 0.69 to 1.09) monthly increase before program entry, compared to a 0.37% (95%CI 0.17 to 0.58) monthly decline afterwards, a drop of 1.26% (*p* < 0.0001, 95%CI 1.08 to 1.44). Entry into the program was associated with a change from weight gain to weight loss. 

### 3.3. Secondary Outcomes

There were significant decreases in mean BMI *z*-score, (2.26 ± 0.33 to 2.2 ± 0.36, *p* < 0.001) and in mean waist circumference (99 ± 15.7 to 97 ± 16, *p* < 0.0001) over the 10 weeks. Fasting insulin decreased from 137 ± 94.8 to 121 ± 83.4 (*p <* 0.001). There was also a trend in reduction of HOMA-IR (4.28 ± 3.02 to 3.77 ± 2.73, *p* = 0.086); however, this was not statistically significant. There was no difference in mean fasting glucose from intake to program evaluation. 

Changes in self-reported physical activity as measured by the PAQ over the course of the 10-week program are presented in Table 3. Total minutes and MET*minutes of exercise increased significantly, as did minutes and MET*minutes of moderate physical activity. Total self-reported physical inactivity decreased by a mean of 330 ± 575 min (*p* < 0.0001).

Mean BYI-II T-Score for Self Concept significantly improved from 46.4 ± 7.86 to 51.5 ± 9.56 (*p* < 0.001). The mean BYI-II T-Score for anxiety significantly decreased from 49.6 ± 8.70 to 45.7 ± 11.80 (*p* = 0.001). Mean BYI-II T-Scores for depression were 47.2 ± 8.31 and 46.2 ± 9.35 before and after the program, respectively, and were not statistically different (*p* = 0.21).

**Table 3:** Seven-day Physical Activity Questionnaire (PAQ) Results *.

	Baseline	Evaluation	Difference	*p*-value
Exercise (min)	309 ± 267	461 ± 425	152 ± 412	0.001
Exercise (MET min)	1955 ± 1748	2942 ± 2675	986 ± 2538	0.001
All Moderate PA (min)	402 ± 285	593 ± 500	191 ± 486	0.001
All Moderate PA (MET min)	2397 ± 1786	3539 ± 2890	1142 ± 2734	0.000
Total physical inactivity (min)	860 ± 663	530 ± 486	−330 ± 575	0.000

**Abbreviations:** MET, metabolic equivalent of energy expanded at rest; PA, physical activity. Data is expressed as mean ± SD; * Subjects who attended >7 sessions.

## 4. Discussion

The CHW-SB family-centered lifestyle intervention is the first naturalistic obesity treatment program cohort to be evaluated in Canada. During the program, participants’ weight gain slowed significantly compared to weight gain observed while waiting for the program to begin. This resulted in a dramatic reduction in the slope of the weight change trajectory from Phase 1 (before program: intake to baseline) to Phase 2 (during program: baseline to evaluation). Furthermore, longitudinal mixed effects regression analysis revealed that more days spent in the program resulted in a greater reduction in weight trajectory. In addition to these encouraging changes, when BMI was standardized for the child’s age and sex by conversion to a *z*-score, we observed that BMI *z-*score was also significantly reduced in participants during the 10-week program. These findings are of utmost clinical significance given recent data demonstrating that impaired glucose tolerance can be reversed if BMI is stabilized and that the probability of impaired glucose tolerance progressing to type 2 diabetes increases if BMI continues to rise [48].

At least 17 other pediatric obesity intervention programs similar to the CHW-SB program exist in Canada; however, to our knowledge, formal evaluation of these programs has not yet been reported [49]. In the United States, Dreimane *et al.* also evaluated a family-centered, weight management program for overweight children [50]. Like the CHW-SB program, this program (Kids N Fitness) was an outpatient hospital-based program for children and their families that utilized exercise and nutrition education and behaviour modification and also included participants developing exercise and nutrition goals during the program. Consistent with our results, children who participated in Kids N Fitness had lower weight velocity and had reduced BMI *z*-scores during the 12-week program compared to before the program started. Furthermore, this study also noted improvements in emotional well-being as measured by a Child Health Questionnaire. Savoye and colleagues [51] also implemented a weight management family-based program called *Bright Bodies* for overweight children and found that after 12 months, participants gained significantly less weight during the program than controls. Like CHW-SB, this program also utilized a behaviour modification component, exercise component, and a nutrition education component focusing on a non-diet approach. In addition, the program evaluated by Savoye *et al.* [51] found that after one year, BMI and body fat were reduced compared to control subjects. These results are also consistent with another urban weight management program evaluation conducted by Evans and colleagues where after six months, BMI *z*-score was found to decrease by 1.2% [52]. In a longer evaluation time-frame, Vignolo *et al.* [53] found that five years after a multi-disciplinary, hospital-based outpatient weight management program similar to the CHW-SB program, children who participated had significant reductions in mean BMI standardized for age and sex (baseline: 4.23 ± 0.71 *versus* 5-year follow-up: 2.74 ± 0.85). It will be important to continue to evaluate *CHW-SB* program to assess whether the positive results reported herein can be sustained over the long-term.

Along with improvements in weight and *z*BMI seen in CHW-SB participants came improvements in some of the metabolic syndrome components. Waist circumference decreased considerably over the relatively short evaluation time period of 10 weeks. This reduction is clinically relevant, since it has previously been shown that abdominal adiposity is a serious risk factor for cardiovascular disease [54]. Furthermore, we observed a decrease in fasting insulin and a trend for a decrease in HOMA-IR. Given that previous studies [55,56] have demonstrated that obesity, and glucose intolerance in childhood have been strongly associated with increased rates of premature death, interventions that improve these parameters are of utmost importance in this population that is still growing and developing. 

The CHW-SB program also resulted in dramatic increases in physical activity. MET*minutes of exercise and moderate physical activity both increased by almost 50% and minutes of physical inactivity decreased by 38% over the 10-week program. In Virginia, TEENS was developed as a weight management program for adolescents with BMI ≥ 95^th ^percentile [52]. Like CHW-SB, TEENS is run as an outpatient program out of a children’s hospital and includes components of medical assessment, nutrition and behavioural modification. In addition, TEENS also involves a rigorous exercise program. In an evaluation of TEENS conducted by Evans *et al.*, it was found that in addition to a decrease in BMI *z*-score, there was a significant improvement in cardiorespiratory fitness; however, unlike *CHW-SB*, this program found a non-significant (*p* = 0.08) improvement in hours of moderate physical activity per week [52].

In addition to the significant improvement in adiposity, metabolic, and physical activity profiles observed in participants, the CHW-SB program also resulted in both clinically significant reductions in self- reported anxiety and increases in self-concept (self-esteem) as measured on the Beck Youth Inventories Second Edition. Our results are consistent with both the results of the Kids N Fitness where, after 12 weeks, participants’ emotional well-being and behaviour improved [50] and with a 5-year evaluation of *Mi Piace Piacermi*, where participants’ emotional and social behaviours improved [53]. As well, similar results were observed in the previous evaluation of the *Shapedown* program where after 15 months, a large improvement in self-esteem was observed both in a general population of overweight children and in a sub-population of obese children with type 2 diabetes [29,30]. These findings are especially important given the vast literature describing that obese children have a greater incidence of internalizing and externalizing problems, lower social competence and reduced self-esteem [57,58,59].

While other North-American weight management programs for overweight children have been successful in the past, the multi-disciplinary, family-centered approach, coupled with group therapy and a comprehensive medical evaluation, makes the CHW-SB program unique [49]. In conjunction with the diverse nature of the program, our program evaluation also included adiposity, biochemical, psychological and physical activity measures, which allowed us to assess multiple aspects of this weight management intervention. Because obese youth have been previously found to have co-morbid psychological and medical complications, it is vital that both our program and evaluation methodology include a comprehensive, multidisciplinary approach. Moreover, by taking advantage of the time that children waited for program commencement (Phase 1) to obtain a natural control for weight changes observed during the program (Phase 2) allowed us to assess the effect of CHW-SB program on the weight trajectory, which was important for accurate analysis of the program’s impact over the short-term.

## 5. Strengths and Limitations

The results of this study must be interpreted within its limitations. The main limitation of this study was that we did not have a standard control group. Nonetheless, we were able to utilize the waiting period before the program began to use each child’s data from baseline to intake as their own “control” for their changes during the program. Having a naturalistic comparison for weight changes that is appropriately matched for age and sex is a strength of this study. The lack of full follow-up is also a limitation in interpreting the effectiveness of this program, as drop-out may be associated with lack of success in meeting program goals. The longitudinal analysis ameliorates this issue but does not avoid it completely. The analyses of changes between visit 1 and visit 10 may reflect bias in the optimistic direction. Additionally, the PAQ only examined participants who attended >7 sessions; thus improvements in physical activity observed in our evaluation may be linked to attendance.

Unfortunately, we were not able to assess whether the CHW-SB program decreased the prevalence of dyslipidemia and hypertension since these parameters were not measured at evaluation. It will be important that future program evaluation include analysis of these parameters to properly assess the programs influence on the metabolic syndrome. Furthermore, our evaluation did not include an assessment of nutrition or an objective assessment of physical activity. Both are important to include in subsequent evaluations to help us understand which behaviours are modified as a result of intervention. 

While we had positive results, they were of modest magnitude, and our evaluation did not include long-term follow-up. Our evaluation was limited because government funding for the CHW-SB program did not include the cost of program evaluation. Future program support should include means for long-term systematic evaluation to assess program effectiveness and ensure that tax-payer dollars are being spent appropriately. Finally, our participation rate of 71% is a strength as it is an improvement on participation rates of 63% reported in a weight intervention program of similar duration in children [50].

## 6. Conclusions

CHW-SB, a government-funded program, is the first obesity treatment program to be evaluated in Canada. While short-term evaluation revealed significant improvements in adiposity, PA, and psychological measures, these data emphasize the need for ongoing evaluation to assess the long-term implications of this unique program and ultimately optimize utilization of governmental resources. 
